# Surface Area Evaluation of Electrically Conductive Polymer-Based Textiles

**DOI:** 10.3390/ma11101931

**Published:** 2018-10-10

**Authors:** Lukas Vojtech, Marek Neruda, Tomas Reichl, Karel Dusek, Cristina de la Torre Megías

**Affiliations:** 1 Department of Telecommunication Engineering, Faculty of Electrical Engineering, Czech Technical University in Prague, 166 27 Prague, Czech Republic; vojtecl@fel.cvut.cz (L.V.); nerudmar@fel.cvut.cz (M.N.); cristinatm07@gmail.com (C.d.l.T.M.); 2 Department of Electrotechnology, Faculty of Electrical Engineering, Czech Technical University in Prague, 166 27 Prague, Czech Republic; dusekk1@fel.cvut.cz

**Keywords:** electrically conductive textiles, polymers, smart textiles, surface area evaluation

## Abstract

In this paper, the surface area of coated polymer-based textiles, i.e., copper and nickel plated woven polyester fabric, copper and acrylic coated woven polyester fabric, and copper and acrylic coated non-woven polyamide fabric, is investigated. In order to evaluate the surface area of the woven fabrics, Peirce’s geometrical model of the interlacing point and measurement using an electron microscope are used. Non-woven fabrics are evaluated using an optical method, handmade method, and MATLAB functions. An electrochemical method, based on the measurement of the resistance between two electrodes, is used for relative comparison of the effective surface area of the coated woven and non-woven fabrics. The experimental results show that the measured and calculated warp lengths do not differ within the standard deviation. The model for the surface area evaluation of the Pierce’s geometrical model for monofilament (non-fibrous) yarns is extended to multifilament yarns and to a uniform sample size. The experimental results show the increasing trend of surface area evaluation using both modeling and electrochemical methods, i.e., the surface area of the copper and acrylic coated woven Polyester fabric (PES) is the smallest surface area of investigated samples, followed by the surface area of the copper and acrylic coated non-woven fabric, and by copper and nickel plated woven PES fabric. These methods can be used for surface area evaluation of coated polymer-based textiles in the development of supercapacitors, electrochemical cells, or electrochemical catalysts.

## 1. Introduction

Electrically conductive textile materials have attracted considerable attention from the scientific community and have found their use in many applications. These materials are used in shielding against electromagnetic radiation, e.g., protective clothing, cable shielding, shielding of high frequency sources, etc. [1,2,3,4,5], in development of textile antennas [6,7,8] and sensors, e.g., textile sensor for Electrocardiography monitoring (ECG), and pressure and humidity sensors [9,10,11], as well as in the development of other electrical components and devices, e.g., supercapacitors, electrochemical cells, electrochemical catalysts, etc. [12,13,14].

There are many ways to improve the parameters of the last mentioned components [15]. The reduction of the internal resistance decreases the thermal loss, increases the capacity of electrochemical cells, and causes a better current distribution. One of the possible ways to reduce the internal resistance is to increase the relative effective surface area in 3-D of the sub-parts of these components (electrodes), i.e., the enlargement of the reaction area upon which the chemical reactions occur. It will result in greater electrochemical interconnection between the electrode and the electrolyte. In electrochemical catalysts, the increase of surface area, i.e., reaction area, causes a larger reaction area to be available during the chemical reaction, e.g., in fuel cells. In supercapacitors, the increase of surface area increases the capacity [15]. 

The surface area, i.e., reaction area, can be increased by using electrically conductive polymer-based textiles. The electrically conductive polymer-based textiles are an interesting class of materials that combine some mechanical properties of polymers with electrical properties typical of metals [16]. This is especially true for conductivity and permanence in applications where the chemical process takes place [15,16]. Additionally, these polymers have become popular because they are lightweight and economical, and have relatively high adjustable electrical conductivity, flexibility, chemical stability, and biocompatibility. The biggest advantage of conductive polymers is their processability [16]. For the future construction and engineering of the mentioned components, it is necessary to know the value of the surface area of their sub-parts. This is typically in the form of a grid, expanded metal, or plate-shaped. However, the shape of the surface area of the electrically conductive polymer-based textile, e.g., coated polymer textile, is not a typical one. The method most commonly used to determine the specific area of a solid porous substance is the Brunaer, Emmett, and Teller method (BET) [17,18]. This method relies on the BET equation for specific isothermal gas adsorption [17]. Although the BET method remains a popular choice for assessing the specific surface areas of nanostructured materials [18], it is speculated that BET does not provide a true representation of the geometric area. It is known that the applicability of the BET method to the porosity characterization is problematic because it is unable to capture complex adsorption mechanisms due to microporous effects [18]. BET regions are highly susceptible to the size of pores, heterogeneity of structures, and adsorbent–adsorbent interactions. There is no simple correlation between BET and geometric surface areas of nanoporous materials [18].

In this paper, three different coated polymer-based textiles, i.e., copper and nickel plated woven polyester (PES) fabric, copper and acrylic coated woven PES fabric, and copper and acrylic coated non-woven polyamide fabric, are tested for the surface area evaluation. The arrangement of the fiber in the woven fabric is investigated using the Peirce’s geometrical model of the interlacing point. This model can be applicable for calculation of the surface area of monofilament (non-fibrous) yarns. The model for surface area evaluation of the multifilament yarns of woven fabric is calculated from the theoretical lengths of yarns, which are obtained by means of an electron microscope. Three methods are used for evaluation of the surface area of non-woven fabrics, i.e., handmade selection method, automatic image conversion to binary method, and threshold selection method. Results are compared with an electrochemical method, which is based on measurement of resistance between two immersed electrodes inside an electrolyte. These methods evaluate the 3-D surface area of the coated polymer-based textiles and can be used in the development of electrical and electrochemical components and devices such as supercapacitors, electrochemical cells, or electrochemical catalysts. The knowledge of surface area evaluation of a conductive 3-D layer of coated textiles can help in adjusting the weight, energy density, and capacity of these future components and equipment.

## 2. Materials and Methods

Three different types of polymer fabrics were tested, i.e., two types of woven fabrics and one type of nonwoven fabric, Table 1. CerexCuIv4 is a copper- and acrylic-coated non-woven fabric with a raw material Polyamide Cerex fabric 36 g/m^2^ and surface resistivity 0.02 ohm/square [19]. RSKCu+Ni is a copper- and nickel-plated woven PES fabric with a plain weave (parachute silk) and a surface resistivity 0.02 ohm/square [20]. RSKCuIv4 is a copper- and acrylic-coated woven PES fabric with a plain weave (parachute silk) and a surface resistivity 0.05 ohm/square [20]. Samples were examined using a scanning electron microscope (Phenom pro X ThermoFisher SCIENTIFIC, Eindhoven, The Netherlands).

### 2.1. Theory of Peirce’s Geometrical Model of the Interlacing Point

**Figure 1:**
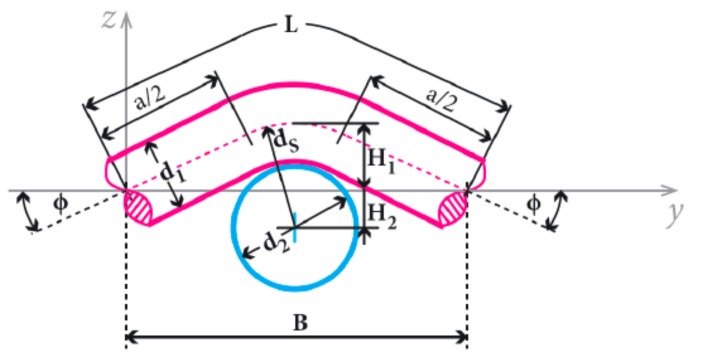
Peirce’s geometrical model of the interlacing point [22]. Legend: *a* is the length of the line connecting the arcs, *d*_1_ is the diameter of the warp, *d*_2_ is the diameter of the weft, *d*_s_ is the sum of radii of the warp and weft yarns, *H*_1_ is the height of warp interlacing wave, *H*_2_ is the height of weft interlacing wave, *B* is the spacing of weft yarns, *Φ* is the warp interlacing angle, and *L* is the length of warp yarn.

The arrangement of the fiber in the fabric can be mathematically described in several ways. The simplest, but fully satisfactory, is the so-called the Peirce’s geometrical model of the interlacing point [21,22] (Figure 1). This model describes the fiber as a system of a regular repeating part of a torus (a body formed by rotating a circle around a straight line) and a cylinder of height *d*. The disadvantage of the Peirce’s geometrical model of the interlacing point is that it neglects the deformation of the fiber caused by forces that act on the fibers, such that shape of the fiber is always circular [21,22]. The main reason for using this model is a satisfactory balance between the efficiency of using it and its accuracy [22].

The relationship between the warp interlacing angle *Φ* and other geometric parameters (spacing of weft yarns *B*, height of the warp interlacing wave *H*_1_, and the sum of radii of the warp and weft yarns *d*_S_) can be expressed using Equations (1) and (2) resulting from the section in the *y*-axis Equation (1) and in the *z*-axis Equation (2) [22]:

(1) $$2\cdot d_{S}\cdot sin\left(\Phi \right)+a\cdot cos\left(\Phi \right)=B$$

(2) $$2\cdot d_{S}\cdot \left[1-cos\left(\Phi \right)\right]+a\cdot sin\left(\Phi \right)=2\cdot H_{1}$$

The sum of the radii of the warp and weft yarns *d*_s_ is calculated using Equation (3), Figure 1:

(3) $$d_{S}=\left(d_{1}+d_{2}\right)/2$$

Combining Equations (1) and (2), a quadratic equation for the warp interlacing angle *Φ* is obtained, as shown in Equation (4):

(4) $$\left[B^{2}+4\cdot {\left(H_{1}-d_{S}\right)}^{2}\right]\cdot cos^{2}\left(\Phi \right)+8\cdot d_{S}\cdot \left(H_{1}-d_{S}\right)\cdot cos\left(\Phi \right)+\left(4\cdot d_{S}^{2}-B^{2}\right)=0$$

The warp interlacing angle *Φ* can be expressed from Equation (4) as in Reference [23], given here as Equation (5):

(5) $$\Phi =arccos\frac{2Md\pm 16r^{2}}{2\left(M^{2}+4r^{2}\right)}$$

where *M* is the spacing of yarns, *r* is the radius of yarns, and *d* is the height of cylinder of yarns.

The height of the cylinder of yarns *d* is then calculated using Equation (6):

(6) $$d=\sqrt{M^{2}-12r^{2}}$$

The theoretical length of the warp *L*_WARP_ and weft *L*_WEFT_ is calculated using Equations (7) and (8):

(7) $$L_{WARP}=2\cdot d_{S}\cdot \Phi +a_{WARP}$$

(8) $$L_{WEFT}=2\cdot d_{S}\cdot \Phi +a_{WEFT}$$

where *a*_WARP_ is the length of the line connecting the arcs in warp, *a*_WEFT_ is the length of the line connecting the arcs in weft.

### 2.2. Surface Area Evaluation Based on Peirce’s Geometrical Model of the Interlacing Point

To calculate the effective surface area of the conductive fabrics, i.e., the surface area that is involved in chemical reactions, the warp and weft lengths of Pierce’s geometrical model of the interlacing point are first determined. The theoretical length of weft and warp are calculated from Equations (7) and (8). The length of the line connecting the arcs, diameter of warp, and diameter of weft are measured by means of an electron microscope, Phenom pro X (ThermoFisher SCIENTIFIC). From Equations (5) and (6), we obtain the warp interlacing angle and weft angle. These angles are substituted into Equations (7) and (8), which gives the theoretical length of the warp and weft. The surface area of the warp and weft is then calculated using Equations (9) and (10):

(9) $$S_{\mathrm{WARP}}=2\pi r_{1}L_{WARP}$$

(10) $$S_{\mathrm{WEFT}}=2\pi r_{2}L_{WEFT}$$

The surface area of the interlacing points is calculated using Equation (11):

(11) $$S_{\mathrm{Inter}\_\mathrm{Point}}=2\pi r_{1}r_{2}$$

The surface area of the investigated Pierce’s geometrical model of the interlacing point of the textile is a sum of the warp and weft surface area, from which the surface area of interlacing points is subtracted (Equation (12)). The obtained value is then divided by two due to the 50% of the depth efficiency of the electrochemical process, which is related to the object used in the fabric structure. This ratio will be different for the electrochemical reaction and electrolyzer construction in practice. For comparison of examined methods, the ratio 50% is chosen, i.e., “visible part of the surface area” during the observation of the structure in the direction of the *z*-axis is considered.

(12) $$S_{\mathrm{Effect}}=(S_{\mathrm{WARP}}+S_{\mathrm{WEFT}})/2\;-\;S_{\mathrm{Inter}\_\mathrm{Point}}$$

Equation (12) can be applicable for the calculation of the surface area of monofilament (non-fibrous) yarns. The surface area of multifilament yarns in warp *S*_1_ is obtained by multiplying the theoretical length of the warp *L_WARP_* and the width in weft *b* and coefficient *K*, which takes into account the amorphousness of the fibers, as seen in Equation (13):

(13) $$S_{1}=L_{\mathrm{WARP}}\cdot b\cdot K$$

where *S*_1_ is the surface area of the multifilament yarns in warp, *b* is the width in weft, and *K* is the coefficient amorphousness of the fibers.

The same procedure as for *S*_1_ is used to calculate the surface area of the multifilament yarns in weft *S*_2_, as seen in Equation (14):

(14) $$S_{2}=L_{\mathrm{WEFT}}\cdot c\cdot K$$

where *c* is the width in warp.

**Figure 2:**
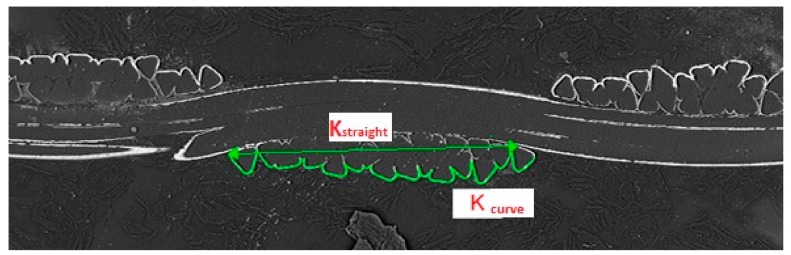
Evaluation of the width of the multifilament yarns *K*_straight_ and the length of coating of multifilament yarns in cross-section *K*_curve_.

The *K* is calculated as the ratio of the width of the multifilament yarns *K*_straight_ and the length of coating of multifilament yarns in cross-section *K*_curve_, which is shown in Figure 2 and given in Equation (15). The difference in the shape of the yarns, i.e., difference between elliptical shape of yarns as assumed by the Pierce’s geometrical model of the interlacing point and the real shape of yarns as shown in Figure 2, is assumed to be negligible. This assumption is verified in Section 3.2 (Table 7) and Section 3.3 (Table 12).

(15) $$K=\frac{K_{\mathrm{curve}}}{K_{\mathrm{straight}}}.$$

where *K*_straight_ is the width of the multifilament yarns and *K*_curve_ is the length of the coating of multifilament yarns in cross-section.

### 2.3. Optical Method for the Non-Woven Fabric Evaluation

#### 2.3.1. Handmade Selection Method

**Figure 3:**
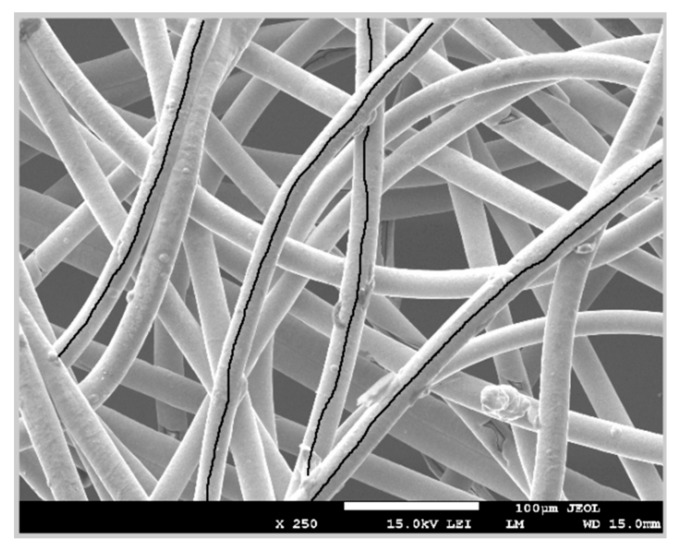
Selection of the fiber lengths by hand.

One of the methods used for surface area evaluation is a time-consuming handmade selection method. It is based on choosing all fibers individually by hand in the obtained picture from the electron microscope image (Figure 3). It illustrates individual fibers and the background, represented by the light and dark areas, respectively.

The white bar that is given at the bottom of the picture is used as a reference value in µm to match the measured length of each fiber. For this reason, the length of this scale bar has to be saved as a variable. The measurement of the scale bar is accomplished by selecting the length of the bar each time the method is executed. It is carried out with the function of MATLAB “imline” [24].

Once the bar length is selected, the diameter of one fiber is selected by hand [24]. We assume the diameter is the same in all fibers. Therefore, fiber selection for measuring the diameter does not have the relevance. To increase accuracy, it is recommended to follow these two recommendations:

- Select the diameter in the upper fibers, which are sharper than the rest.
- Choose fibers that are directly in contact with the background since the boundaries of these fibers can be differentiated more easily.

After this, a line that covers the whole length of each fiber is traced to obtain the fiber length. It is important to take into account that the lines are determined by hand, using the function “imfreehand” of MATLAB [25]. Lines are drawn in the center of every fiber with the highest precision the user can perform.

Next, the length of the fiber and the diameter are compared with the scale bar length and the area is calculated using the known mathematical equation for the volume of a cylinder, i.e., half of 3-D surface area is calculated [26]. Finally, the total area is the sum of the area of all fibers.

As it can be appreciated in the described method, accuracy of the length measurements depend on the user. At least lengths referring to the bar and diameter are straight lines and are taken only once, but every fiber length is completely determined by hand, which increases the error. This error can be mitigated by creating a function that calculates the exact points of the center of the yarn. It must also be taken into account that the mistake that may appear in the selection of fibers that are missing in each step, i.e., the user can forget some fibers or parts of them that seem part of background.

#### 2.3.2. Automatic Image Conversion to Binary

Another method for the surface area evaluation of non-woven fabrics is the conversion of the obtained microscope picture of the sample to a binary image. It consists of changing all fibers to white and the background to black to differentiate clearly which parts of the image are conductive.

The same sample is used for the surface area evaluation as described in the previous section. As per the previous method, the length of scale bar is measured by hand. However, the used standard MATLAB function “imtool” provides the possibility to check the value of the pixels, 0 (black) or 255 (white) [27]. When the user selects the length manually, they check the pixel value at the same time. If it is white, the user starts to measure the length and stops the measurement when they see the change from 255 to 0.

The procedure of the diameter measurement is the same as the bar measurement. The user can appreciate how the intensity of gray varies, since white values of pixels are higher than black values, therefore the user knows when to start and stop the measurement. As explained in the previous section, the diameter is selected according to the two described recommendations.

**Figure 4:**
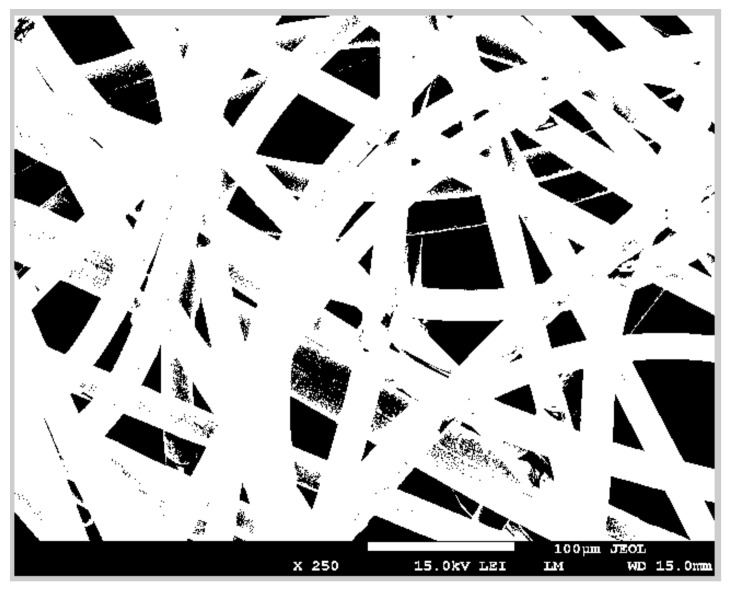
Binary image.

After that, the original image is converted to a binary image (Figure 4). The input image is converted to a grayscale format (if it is not already an intensity image), and then to binary. This procedure is directly performed using the function “im2bw (I, 0.5)”.

Next, the user selects the part of image that contains fibers, and pixels in white are counted [28]. In case two consecutive pixels are white, the distance between them is calculated and saved into a variable. This variable, and also the diameter, are compared with the scale bar, and then the area is calculated using the same equation as in the previous method.

As shown in Figure 4, when the image is converted to binary, not all the fibers are completely white, which is the main cause of the error in this method. In this case the improved function consists of delimiting the boundaries of all fibers and fills the pixels inside these limits. Therefore, pixels inside fibers that are not in white using function “im2bw” will be converted using this improved function. The other cause comes from the user when the user takes measurements of the scale bar and diameter. Another source of error appears when the image is cropped. The user can make a mistake if they select a smaller size of the required image [28].

#### 2.3.3. Threshold Selection Method

The third method used for evaluating the surface area of non-woven fabrics is the transformation of the color of the background using thresholding. This method is based on finding the appropriate threshold in the gray scale to change the background pixels to black. Therefore, the fibers can be seen more clearly in the image. The same image sample is used as in the previous cases (Figure 3). Measurement of the scale bar and diameter is performed according to the same procedure as in the previous method, i.e., the function “imtool” is used. 

**Figure 5:**
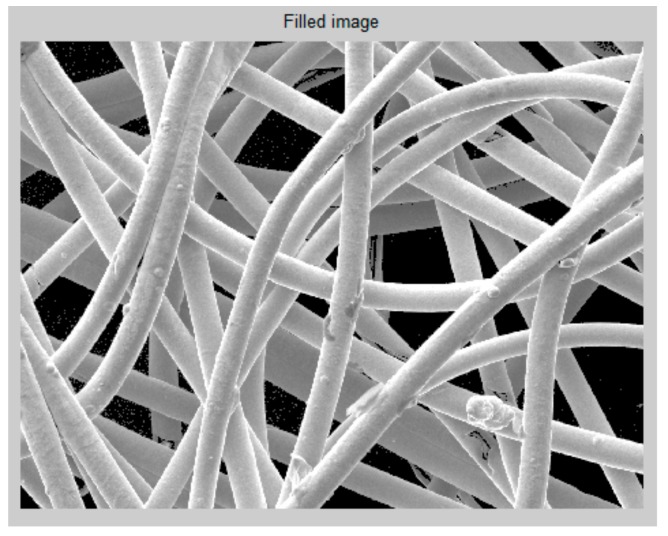
Filled image using a threshold to change the background color.

When the measured values of the length of the scale bar and diameter are saved, the image is cropped to work only with the part that contains the fibers. The difference between this method and previous one is that the original image is not converted to binary. In this case, a threshold is used whose value can be changed to achieve the best results. The background color is changed to black to check the amount of pixels that are considered background (Figure 5).

As can be seen from Figure 5, some parts inside the fibers are painted in black because they are darker, such that the threshold condition is met due to deformation of the fiber. On the contrary, not all parts of the background are painted in black because all backgrounds do not have the same color or the same intensity of black color. This is a major error for this method and should also be considered a user-side error as described in the previous method. In conclusion, the preferred method is the threshold selection method, due to its higher accuracy and lower assistance requirement from the user.

### 2.4. Experimental Electrochemical Method for Surface Area Evaluation

The surface area of the coated woven and non-woven fabrics can be also evaluated using an electrochemical method, based on the measurement of the resistance between two electrodes. This method is effective, i.e., easy to use, for the relative comparison of surface area values, and is not suitable for a total surface area evaluation for several reasons. The contact resistance error, i.e., systematic error, is eliminated by comparative measurement. The electrode polarization errors are eliminated by using an alternating current and a small current (i < 100 μA). Chemical changes of the electrolyte are eliminated by the low conductivity of the electrolyte, the small current used, and by the high volume of electrolyte. The conductivity of the metal coating of textiles is much higher than the conductivity of the electrolyte (H_2_O), therefore the voltage drop in the electrolyte is much higher than the voltage drop on the coated fabric, and thus different coatings of the textiles can be neglected. The electrodes are placed in a plastic holder that is immersed into the electrolyte (H_2_O, with temperature 25 °C). The first (base) electrode is fixed in a stable position in a plastic holder. The material of this electrode is a Cu plate, i.e., fully copper-plated printed circuit board with a surface area of copper 3.06 × 10^−3^ m^2^. It is chosen because of a good surface flatness and negligible thickness of the conductive copper layer. The following four types of the second electrode are used:

- Cu plate (same sample properties as the first (base) electrode)
- CerexCuIv4
- RSKCu+Ni
- RSKCuIv4

**Figure 6:**
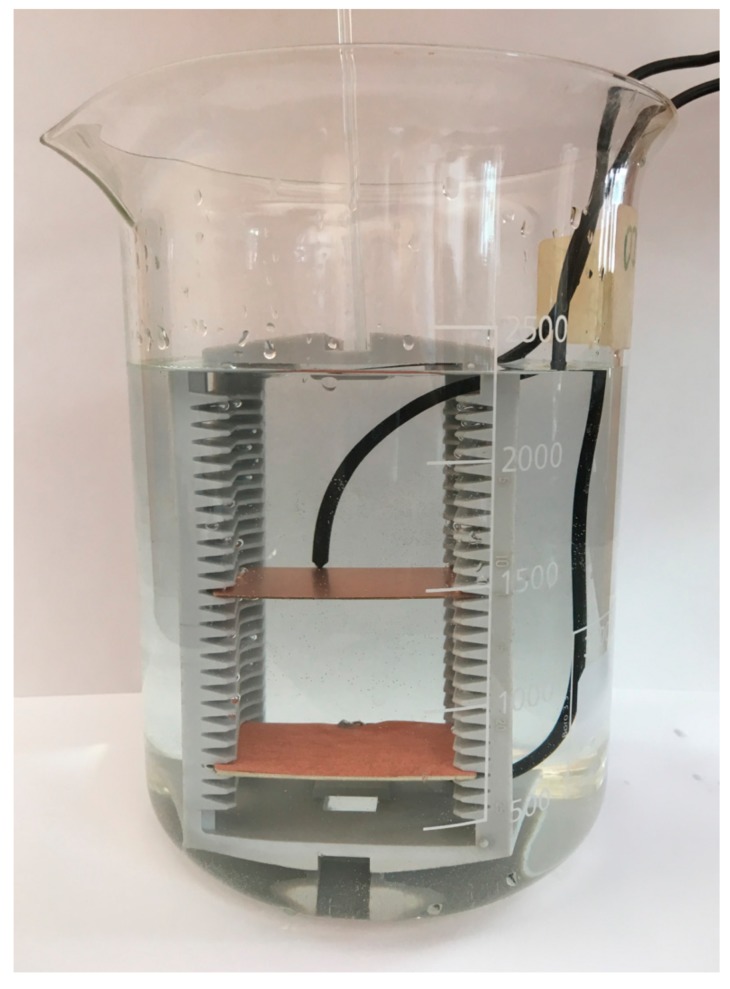
Immersed plastic holder with electrodes inside the electrolyte to give a measurement of the resistance between two electrodes.

In case of fabric samples, the samples were stuck on non-conductive plate using double-sided adhesive tape. The size of the surface area of all electrodes was the same, i.e., 3.06 × 10^−3^ m^2^. An immersed plastic holder with electrodes inside the electrolyte is shown in Figure 6. The distance between the electrodes is changed during the measurement of resistance between electrodes. The resistance is measured by Battery Analyzer, 60 V Battery Analyzer BA6010 (BK Precision, Yorba Linda, CA, USA) at 1 × 10^3^ Hz for three different distances between electrodes, i.e., 0.03, 0.04, and 0.05 m.

Comparison of the surface area of samples was based on the electrolyte resistivity constant and the distance between the electrodes:

(16) R·S=ρ·l=const.

where *R* is the measured resistance, *S* is the surface area, *ρ* is the resistivity of electrolyte, and *l* is the distance between electrodes.

Equation (16) shows the comparison of the surface area of samples was relative, i.e., it identifies the larger or smaller surface area in comparison with other samples. This comparative measurement method eliminates systematic errors.

## 3. Results and Discussion

### 3.1. Surface Area Evaluation of CerexCuIv4

**Figure 7:**
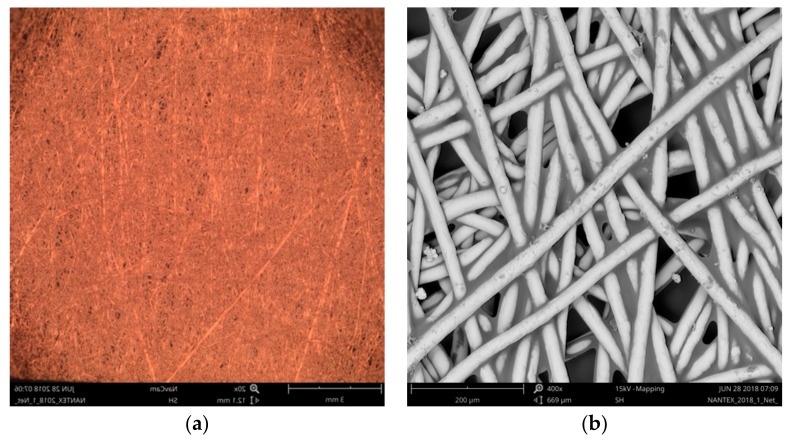
A sample of CerexCuIv4 (coated non-woven fabric) (**a**), and detail of the sample structure (**b**).

The surface area of CerexCuIv4, i.e., coated non-woven fabric, was evaluated by the optical method described in Section 2.3. Figure 7 shows a detail of a sample, which was used for the calculation of the effective surface area. The results are shown for a handmade selection method in Table 2, for the automatic image conversion to binary method in Table 3, and for the threshold selection method in Table 4. The surface area evaluated by the handmade selection method was 2.67 × 10^−7^ ± 1.89 × 10^−8^ m^2^, by automatic image conversion to binary method was 2.30 × 10^−7^ ± 1.49 × 10^−8^ m^2^, and using the threshold selection method was 2.70 × 10^−7^ ± 1.60 × 10^−8^ m^2^.

### 3.2. Surface Area Evaluation of RSKCuIv4

**Figure 8:**
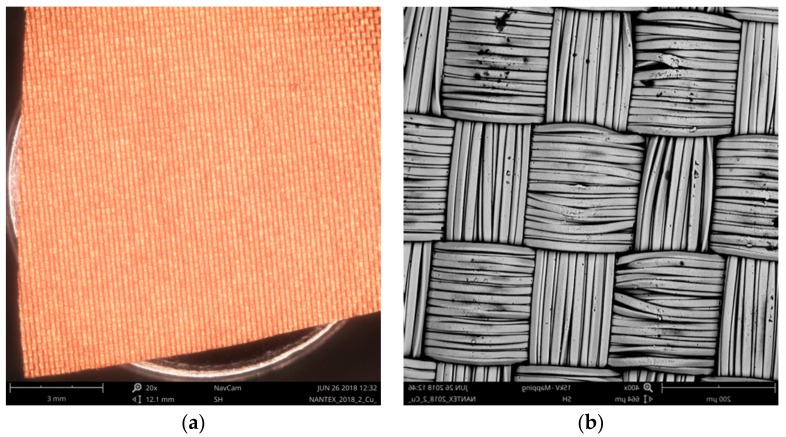
A sample of RSKCuIv4 (**a**), and detail of the sample structure (**b**).

The surface area of RSKCuIv4 was evaluated using the method based on Peirce’s geometrical model of the interlacing point described in Section 2.1 and Section 2.2 (Figure 8).

**Figure 9:**
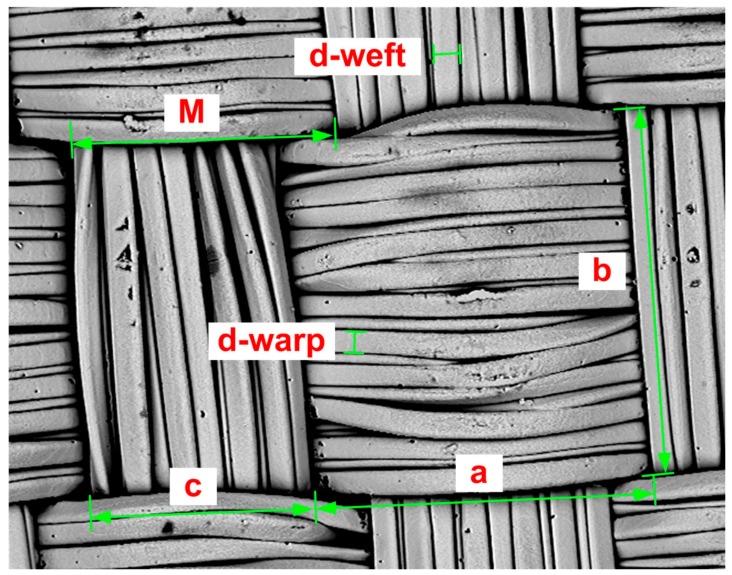
Measurement of input parameters for the theoretical length of the warp and weft calculation for the sample RSKCuIv4.

**Figure 10:**
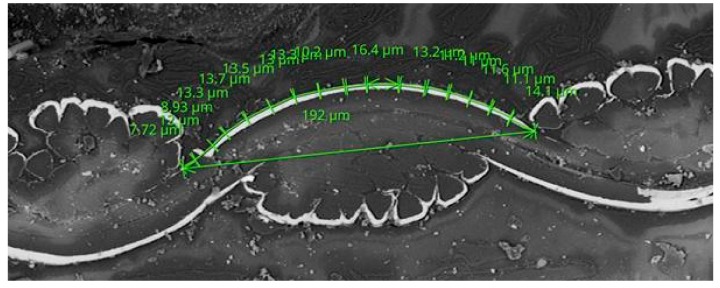
Measurement of the length of the warp for the sample RSKCuIv4.

Figure 9 shows the measurement of input parameters for the theoretical length of the warp and weft calculation using Equations (7) and (8). The length of the line connecting the arcs in warp and weft are marked *a* and *b*, respectively. The sum of the radii of the warp and weft yarns *d*_s_ was obtained from measurement of diameters of the warp and weft yarns, marked *d-warp* and *d-weft*, respectively. The warp interlacing angle *Φ* was obtained from measurement of the spacing of yarns, marked *M*; from calculation of the height of cylinder of yarns, Equation (6), i.e., parameters *M*, *d-warp*, and *d-weft* were considered; and from calculation of the radius of yarns, i.e., parameters *d-warp* and *d-weft* were considered. The theoretical length of the warp *L*_WARP_CALC_ and *L*_WEFT_CALC_ was then calculated (Table 5). The length of the warp *L*_WARP_MEAS_ was also measured (Figure 10 and Table 6). 

The theoretical warp length *L*_WARP_CALC_ was 2.01 × 10^−4^ ± 3.00 × 10^−6^ m and the measured warp length *L*_WARP_MEAS_ was 2.07 × 10^−4^ ± 3.09 × 10^−6^ m. The ratio of *L*_WARP_CALC_ and *L*_WARP_MEAS_ is presented in Table 7. It shows the difference in the units of percentage, i.e., the deformation of the yarns could be neglected, thus the circular shape of cross-section of warp and weft yarns of the Peirce’s geometrical model of the interlacing point could be used. The theoretical warp lengths *L*_WARP_CALC_ and *L*_WEFT_CALC_ were inserted in the Equations (9) and (10) to find *S*_WARP_ and *S*_WEFT_, respectively. The surface area of the investigated Pierce’s geometrical model of the sample was then obtained using Equation (12). The surface area of the investigated multifilament model of the sample was then obtained using Equations (13) and (14) (Table 8).

### 3.3. Surface Area Evaluation of RSKCu+Ni

**Figure 11:**
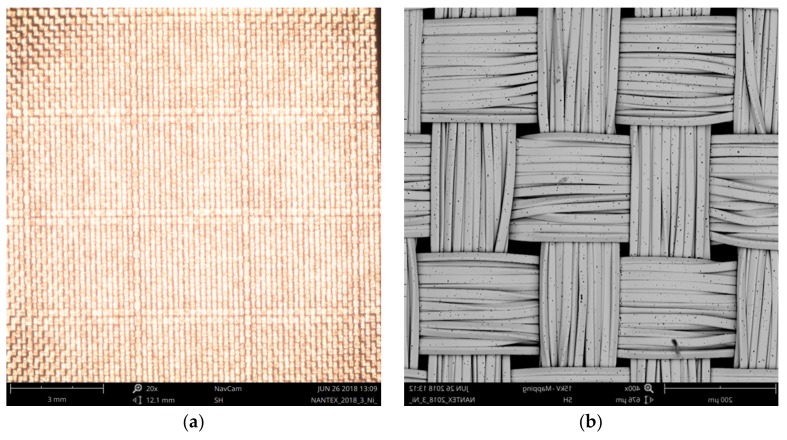
A sample of RSKCu+Ni (**a**), and detail of the sample structure (**b**).

Surface area of RSKCu+Ni was evaluated using the method based on Peirce’s geometrical model of the interlacing point described in Section 2.1 and Section 2.2 (Figure 11).

**Figure 12:**
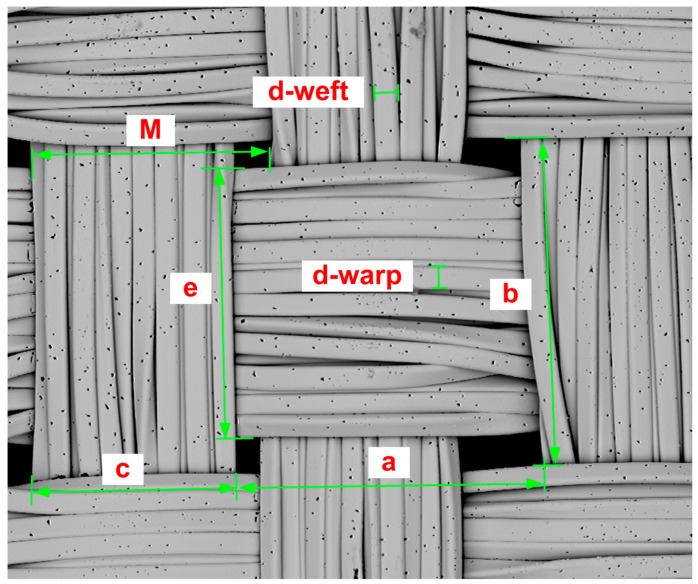
Measurement of input parameters for the theoretical length of the warp and weft calculation for the sample RSKCu+Ni.

**Figure 13:**
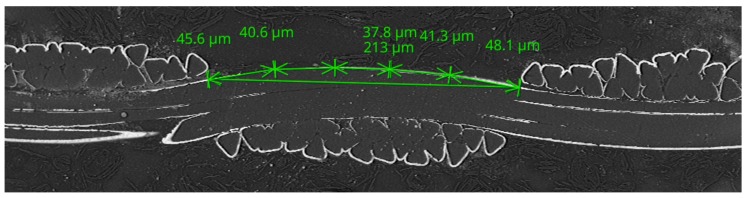
Measurement of the length of the warp for the sample RSKCu+Ni.

Figure 12 shows the measurement of the input parameters for the theoretical length of the warp and weft calculation using Equations (7) and (8). The length of the line connecting the arcs in warp and weft are marked *a* and *b*, respectively. The sum of the radii of the warp and weft yarns *d*_s_ was obtained from the measurement of diameters of the warp and weft yarns, marked *d-warp* and *d-weft*, respectively. The warp interlacing angle *Φ* was obtained from measurement of the spacing of yarns, marked *M*; from calculation of the height of cylinder of yarns, Equation (6), i.e., parameters *M*, *d-warp* and *d-weft* were considered; and from calculation of the radius of yarns, i.e., parameters *d-warp* and *d-weft* were considered (Table 9). The theoretical length of the warp *L*_WARP_CALC_ and the weft *L*_WEFT_CALC_ was then calculated (Table 10). The length of the warp *L*_WARP_MEAS_ was also measured (Figure 13 and Table 11).

The theoretical warp length *L*_WARP_CALC_ was 2.10 × 10^−4^ ± 4.58 × 10^−6^ m and the measured warp length *L*_WARP_MEAS_ was 2.14 × 10^−4^ ± 3.00 × 10^−7^ m. The ratio of *L*_WARP_CALC_ and *L*_WARP_MEAS_ is presented in Table 12. It also shows the difference in the units of percentage, i.e., the deformation of the yarns could be neglected, thus the circular shape of cross-section of warp and weft yarns of the Peirce’s geometrical model of the interlacing point could be used. The theoretical warp lengths *L*_WARP_CALC_ and *L*_WEFT_CALC_ were inserted into Equations (9) and (10) to find *S*_WARP_ and *S*_WEFT_, respectively. The surface area of the investigated Pierce’s geometrical model of the sample *S*_Effect_ was then obtained using Equation (12). The surface area of the investigated multifilament model of the sample RSKCu+Ni was then obtained using Equations (13) and (14) (Table 13). 

### 3.4. Relative Comparison of Surface Areas of Samples Using the Electrochemical Method

**Figure 14:**
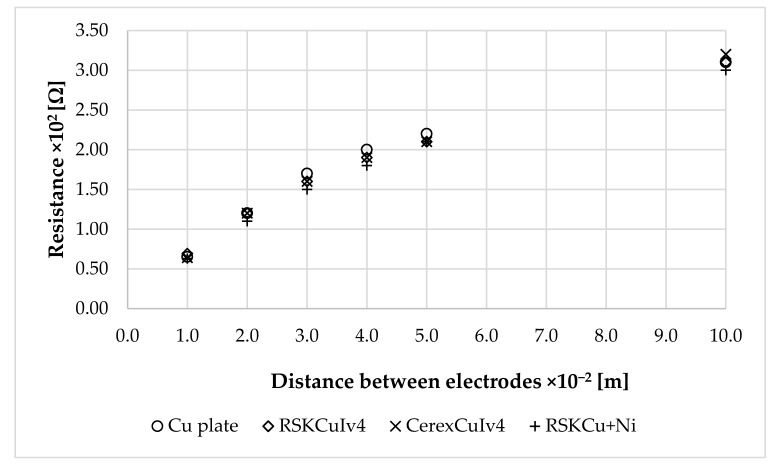
Measurement results of the resistance values in relation to the distance between electrodes.

The surface area of the Cu plate, RSKCuIv4, CerexCuIv4, and RSKCu+Ni was evaluated using the comparative measurement method described in Section 2.4. Table 14 and Figure 14 show measurement results of the resistance between the first (base) electrode, i.e., Cu plate, and the second electrode, i.e., Cu plate, RSKCuIv4, CerexCuIv4, and RSKCu+Ni. The resistance results were inserted into Equation (16) and surface areas were obtained (Table 14).

Resistance values increased with the increased distance between electrodes. This corresponded with the theory as shown in Equation (16), i.e., the surface area was obtained for each sample. The largest surface area was obtained for RSKCu+Ni, followed by CerexCuIv4. The lowest surface area was obtained for RSKCuIv4.

### 3.5. Summary of the Surface Area Evaluation

The theoretical warp length *L*_WARP_CALC_ was 2.01 × 10^−4^ ± 3.00 × 10^−6^ m and the measured warp length *L*_WARP_MEAS_ was 2.07 × 10^−4^ ± 3.09 × 10^−6^ m for the RSKCuIv4. The theoretical warp length *L*_WARP_CALC_ was 2.10 × 10^−4^ ± 4.58 × 10^−6^ m and the measured warp length *L*_WARP_MEAS_ was 2.14 × 10^−4^ ± 3.00 × 10^−7^ m for the RSKCu+Ni. The measured and the calculated values did not differ from each other within the standard deviation, and the theoretical warp lengths *L*_WARP_CALC_ and *L*_WEFT_CALC_ were inserted into Equations (9) and (10) to find the surface area of the warp *S*_WARP_ and weft *S*_WEFT_. The surface area of the investigated Pierce’s geometrical model *S*_effect_ for monofilament (non-fibrous) yarns was then calculated for RSKCuIv4 to be 6.73 × 10^−15^ ± 1.30 × 10^−16^ m^2^ and 7.11 × 10^−15^ ± 1.60 × 10^−16^ m^2^ for RSKCu+Ni. The surface area of multifilament yarns in warp *S*_1_ and weft *S*_2_ was determined for RSKCuIv4 to be *S*_1_ = 6.50 × 10^−14^ ± 1.03 × 10^−15^ m2 and *S*_2_ = 4.74 × 10^−14^ ± 1.03 × 10^−15^ m^2^, and *S*_1_ = 4.88 × 10^−14^ ± 1.07 × 10^−15^ m^2^ and *S*_2_ = 4.30 × 10^−14^ ± 2.60 × 10^−16^ m^2^ for RSKCu+Ni.

The surface area of Cu plate, RSKCuIv4, CerexCuIv4, and RSKCu+Ni was evaluated using the comparative calculation. The results from the individual surface area of the presented models of samples *S*_1_ and *S*_2_ were recalculated to a uniform surface area *S_default_* 3.06 × 10^−3^ m^2^ using Equation (17) (Table 15).

(17) $$S_{Surface\_area\_model}=\left(S_{1}+S_{2}\right)*\frac{S_{default}}{S_{background}}.$$

where *S_default_*is the surface area about size 3.06 × 10^−3^ m^2^ and *S_background_* is the surface area under of Peirce’s geometrical model of the interlacing point of individual samples.

**Figure 15:**
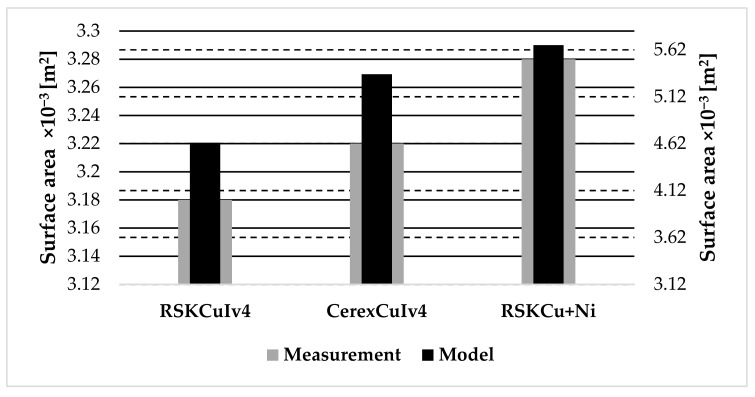
Comparison of modeling and measurement results of the surface area of samples.

Modeling results of the surface area of samples shown in Table 15 were compared with the measurement results shown in Table 14 (Figure 15).

The surface area of RSKCuIv4 was evaluated to be *S*_model_RSKCuIv4_ = 4.63 × 10^−3^ ± 3.62 × 10^−5^ m^2^ and *S*_measurement_RSKCuIv4_ = 3.18 × 10^−3^ ± 4.50 × 10^−5^ m^2^. The surface area of CerexCuIv4 was evaluated to be *S*_model_CerexCuIv4_ = 5.36 × 10^−3^ ± 3.54 × 10^−5^ m^2^ and *S*_measurement_CerexCuIv4_ = 3.22 × 10^−3^ ± 8.22 × 10^−5^ m^2^. The surface area of RSKCu+Ni was evaluated to be *S*_model_RSKCu+Ni_ = 5.67 × 10^−3^ ± 5.23 × 10^−5^ m^2^ and *S*_measurement_RSKCu+Ni_ = 3.28 × 10^−3^ ± 7.48 × 10^−5^ m^2^. The modeling results for the surface area evaluations of the samples showed an increasing trend, i.e., the surface area of the RSKCuIv4 was the smallest surface area of investigated samples, followed by the surface area of the CerexCuIv4, and by RSKCu+Ni. This increasing trend, i.e., the surface area of the RSKCuIv4 was the smallest one, followed by CerexCuIv4 and by RSKCu+Ni, was also shown using the experimental electrochemical method. Both the modeled and the measured surface areas were larger than the uniform surface area, i.e., the sample Cu plate, which corresponded to the theory that the coated polymer-based textiles have a larger surface area than the standard metal plate. The measurement results were of the same order as the modeling results, but showed higher values. This was caused by the used method, which is suitable only for the relative comparison of surface area values as described in Section 2.4.

## 4. Conclusions

Calculation of the surface area of coated woven and non-woven textiles finds its application in development of electrical and electrochemical components and devices such as supercapacitors, electrochemical cells, and electrochemical catalysts. In this paper, several methods for the real effective surface area evaluation of coated woven and non-woven polymer-based textiles were presented. The proposed model for woven textiles was based on Pierce’s geometrical model and on the evaluation of samples by optical methods. The evaluation of non-woven textiles was performed using optical methods and image processing methods. All these proposed models were compared with an experimental electrochemical method for surface area evaluation measurement. The experimental results confirmed the results obtained from modeling. The results show that the largest effective surface area was obtained for copper- and nickel-plated woven polyester fabric and the lowest was obtained for copper- and acrylic-coated woven polyester fabric.

## Figures and Tables

**Table 1:** Specifications of the used commercial polymer coated fabrics [20].

Material as Named by the Producer/Producer	Description	Surface Resistivity	Note
CerexCuIv4/LORIX Ltd., Budapest, Hungary	copper + acrylic coated non-woven fabric	max avg. 0.02 ohm/square	Raw material: Polyamide Cerex fabric 36 g/m^2^
RSKCu+Ni/LORIX Ltd.,	copper + nickel plated woven PES fabric	max avg. 0.02 ohm/square	Weave: plain weave
Budapest, Hungary			(parachute silk)
RSKCuIv4/LORIX Ltd., Budapest, Hungary	copper + acrylic coated woven PES fabric	max avg. 0.05 ohm/square	Weave: plain weave
			(parachute silk)

**Table 2:** Results for the handmade selection method.

Parameter	Average ^1^
Scale bar (pxs)	2.66 × 10^2^
Diameter (pxs)	5.82 × 10^1^
Surface area (m^2^)	2.67 × 10^−7^
Surface area standard deviation *σ* (m^2^)	1.89 × 10^−8^

^1^ Number of measurements: 10.

**Table 3:** Results for automatic image conversion to binary method.

Parameters	Average ^1^
Scale bar (pxs)	2.66 × 10^2^
Diameter (pxs)	5.61 × 10^1^
Surface area (m^2^)	2.30 × 10^−7^
Surface area standard deviation *σ* (m^2^)	1.49 × 10^−8^

^1^ Number of measurements: 10.

**Table 4:** Results for threshold selection method.

Parameters	Average ^1^
Scale bar (pxs)	2.66 × 10^2^
Diameter (pxs)	5.65 × 10^1^
Surface area (m^2^)	2.70 × 10^−^^7^
Surface area standard deviation *σ* (m^2^)	1.60 × 10^−^^8^

^1^ Number of measurements: 10.

**Table 5:** The measurement results of input parameters *a*, *b*, *c*, *M*, *d-warp*, *d-weft*, *L*_WARP_CALC_, and *L*_WEFT_CALC_-RSKCuIv4.

Meas.	*a*	*b*	*c*	*M*	*d-warp*	*d-weft*	*L*_WARP_CALC_	*L*_WEFT_CALC_
No.	(m)	(m)	(m)	(m)	(m)	(m)	(m)	(m)
1	2.01 × 10^−4^	2.12 × 10^−4^	1.45 × 10^−4^	1.55 × 10^−4^	1.08 × 10^−5^	1.02 × 10^−5^	2.03 × 10^−4^	2.13 × 10^−4^
2	2.03 × 10^−4^	2.12 × 10^−4^	1.44 × 10^−4^	1.58 × 10^−4^	1.04 × 10^−5^	1.00 × 10^−5^	2.04 × 10^−4^	2.13 × 10^−4^
3	2.04 × 10^−4^	2.12 × 10^−4^	1.45 × 10^−4^	1.58 × 10^−4^	1.08 × 10^−5^	1.08 × 10^−5^	2.06 × 10^−4^	2.13 × 10^−4^
4	2.02 × 10^−4^	2.17 × 10^−4^	1.47 × 10^−4^	1.60 × 10^−4^	1.08 × 10^−5^	1.05 × 10^−5^	2.03 × 10^−4^	2.18 × 10^−4^
5	2.00 × 10^−4^	2.22 × 10^−4^	1.46 × 10^−4^	1.59 × 10^−4^	1.02 × 10^−5^	1.09 × 10^−5^	2.01 × 10^−4^	2.23 × 10^−4^
6	2.00 × 10^−4^	2.23 × 10^−4^	1.47 × 10^−4^	1.61 × 10^−4^	1.03 × 10^−5^	1.02 × 10^−5^	2.01 × 10^−4^	2.24 × 10^−4^
7	1.97 × 10^−4^	2.25 × 10^−4^	1.46 × 10^−4^	1.59 × 10^−4^	1.09 × 10^−5^	1.00 × 10^−5^	1.98 × 10^−4^	2.26 × 10^−4^
8	1.96 × 10^−4^	2.23 × 10^−4^	1.44 × 10^−4^	1.59 × 10^−4^	1.08 × 10^−5^	1.01 × 10^−5^	1.97 × 10^−4^	2.24 × 10^−4^
9	1.95 × 10^−4^	2.20 × 10^−4^	1.49 × 10^−4^	1.60 × 10^−4^	1.03 × 10^−5^	1.02 × 10^−5^	1.96 × 10^−4^	2.21 × 10^−4^
10	1.97 × 10^−4^	2.17 × 10^−4^	1.45 × 10^−4^	1.62 × 10^−4^	1.08 × 10^−5^	1.03 × 10^−5^	1.98 × 10^−4^	2.18 × 10^−4^
Average	2.00 × 10^−4^	2.18 × 10^−4^	1.46 × 10^−4^	1.59 × 10^−4^	1.06 × 10^−5^	1.03 × 10^−5^	2.01 × 10^−4^	2.19 × 10^−4^
Stand. deviation *σ*	2.90 × 10^−6^	4.80 × 10^−6^	1.47 × 10^−6^	1.80 × 10^−6^	3.00 × 10^−7^	3.00 × 10^−7^	3.00 × 10^−6^	4.70 × 10^−6^

**Table 6:** The measurement results of the length of the warp *L_WARP_MEAS_* and the length of the line connecting the arcs in warp *a*-RSKCuIv4.

Meas. No.	Measured Parts of the arc						*L*_warp_MEAS_	*a*
	(m)						(m)	(m)
1	7.70 × 10^−6^	1.20 × 10^−5^	8.90 × 10^−6^	1.33 × 10^−5^	1.37 × 10^−5^	1.35 × 10^−5^	2.04 × 10^−4^	1.92 × 10^−4^
	1.30 × 10^−5^	1.33 × 10^−5^	1.02 × 10^−5^	1.44 × 10^−5^	1.32 × 10^−5^	1.12 × 10^−5^		
	1.10 × 10^−5^	1.16 × 10^−5^	1.16 × 10^−5^	1.11 × 10^−5^	1.41 × 10^−5^	-		
2	1.00 × 10^−5^	1.30 × 10^−5^	7.00 × 10^−6^	1.20 × 10^−5^	1.10 × 10^−5^	1.40 × 10^−5^	2.06 × 10^−4^	1.93 × 10^−4^
	1.30 × 10^−5^	1.12 × 10^−5^	1.05 × 10^−5^	1.70 × 10^−5^	1.40 × 10^−5^	1.30 × 10^−5^		
	1.05 × 10^−5^	1.15 × 10^−5^	1.30 × 10^−5^	1.30 × 10^−5^	1.25 × 10^−5^	-		
3	1.20 × 10^−5^	1.10 × 10^−5^	1.05 × 10^−5^	1.30 × 10^−5^	1.40 × 10^−5^	1.38 × 10^−5^	2.05 × 10^−4^	1.96 × 10^−4^
	1.25 × 10^−5^	1.05 × 10^−5^	9.50 × 10^−6^	1.45 × 10^−5^	1.40 × 10^−5^	1.09 × 10^−5^		
	1.02 × 10^−5^	1.10 × 10^−5^	1.12 × 10^−5^	1.35 × 10^−5^	1.30 × 10^−5^	-		
4	1.40 × 10^−5^	1.10 × 10^−5^	1.35 × 10^−5^	1.10 × 10^−5^	1.25 × 10^−5^	1.27 × 10^−5^	2.06 × 10^−4^	1.97 × 10^−4^
	1.27 × 10^−5^	1.01 × 10^−5^	1.37 × 10^−5^	1.27 × 10^−5^	1.13 × 10^−5^	1.15 × 10^−5^		
	1.05 × 10^−5^	9.00 × 10^−6^	1.22 × 10^−5^	1.35 × 10^−5^	1.22 × 10^−5^	-		
5	1.30 × 10^−5^	1.25 × 10^−5^	1.17 × 10^−5^	1.20 × 10^−5^	1.27 × 10^−5^	1.30 × 10^−5^	2.13 × 10^−4^	1.98 × 10^−4^
	1.40 × 10^−5^	1.36 × 10^−5^	1.90 × 10^−5^	1.28 × 10^−5^	1.30 × 10^−5^	9.00 × 10^−6^		
	1.12 × 10^−5^	1.10 × 10^−5^	1.28 × 10^−5^	1.17 × 10^−5^	9.70 × 10^−6^	-		
Average	-	-	-	-	-	-	2.07 × 10^−4^	1.95 × 10^−4^
Stand. deviation *σ*	-	-	-	-	-	-	3.09 × 10^−6^	2.30 × 10^−6^

**Table 7:** The comparison of measurement and calculated results of the length of the warp-RSKCuIv4.

Meas.	*L*_warp_MEAS_	*L*_warp_CALC_	*L*_warp_MEAS_/*L*_warp_CALC_
No.	(m)	(m)	(%)
1	2.04 × 10^−4^	2.02 × 10^−4^	100.99
2	2.06 × 10^−4^	2.04 × 10^−4^	100.98
3	2.05 × 10^−4^	2.05 × 10^−4^	100.00
4	2.06 × 10^−4^	2.03 × 10^−4^	101.48
5	2.13 × 10^−4^	2.01 × 10^−4^	105.97
Average	2.07 × 10^−4^	2.03 × 10^−4^	101.88
Stand. deviation *σ*	3.09 × 10^−6^	1.43 × 10^−6^	2.10

**Table 8:** The modeling results for RSKCuIv4.

Parameter	Average	Standard Deviation
*L*_WARP_CALC_ (m)	2.01 × 10^−4^	3.00 × 10^−6^
*L*_WEFT_CALC_ (m)	2.19 × 10^−4^	4.70 × 10^−6^
*S*_WARP_ (m^2^)	6.70 × 10^−15^	1.90 × 10^−16^
*S*_WEFT_ (m^2^)	7.11 × 10^−15^	2.40 × 10^−16^
*S*_Inter_point_ (m^2^)	1.7 × 10^−16^	1.00 × 10^−17^
*S*_Effect_ (m^2^)	6.73 × 10^−15^	1.30 × 10^−16^
*S1*_WARP_Multi_ (m^2^)	6.50 × 10^−14^	1.03 × 10^−15^
*S2*_WEFT_Multi_ (m^2^)	4.74 × 10^−14^	1.03 × 10^−15^

**Table 9:** The measurement results of input parameters *a*, *b*, *c, e*, M, *d-warp*, and *d-weft*-RSKCu+Ni.

Meas.	*a*	*b*	*c*	*e*	*M*	*d-warp*	*d-weft*
No.	(m)	(m)	(m)	(m)	(m)	(m)	(m)
1	1.99 × 10^−4^	2.38 × 10^−4^	1.46 × 10^−4^	1.76 × 10^−4^	1.74 × 10^−4^	1.05 × 10^−5^	1.02 × 10^−5^
2	2.07 × 10^−4^	2.34 × 10^−4^	1.41 × 10^−4^	1.83 × 10^−4^	1.72 × 10^−4^	1.03 × 10^−5^	1.00 × 10^−5^
3	2.07 × 10^−4^	2.39 × 10^−4^	1.53 × 10^−4^	1.80 × 10^−4^	1.70 × 10^−4^	1.01 × 10^−5^	1.05 × 10^−5^
4	2.06 × 10^−4^	2.39 × 10^−4^	1.44 × 10^−4^	1.99 × 10^−4^	1.69 × 10^−4^	1.03 × 10^−5^	1.01 × 10^−5^
5	2.06 × 10^−4^	2.39 × 10^−4^	1.41 × 10^−4^	1.93 × 10^−4^	1.69 × 10^−4^	1.03 × 10^−5^	1.01 × 10^−5^
6	2.08 × 10^−4^	2.39 × 10^−4^	1.48 × 10^−4^	1.93 × 10^−4^	1.67 × 10^−4^	1.03 × 10^−5^	1.02 × 10^−5^
7	2.10 × 10^−4^	2.38 × 10^−4^	1.51 × 10^−4^	1.91 × 10^−4^	1.66 × 10^−4^	1.05 × 10^−5^	1.09 × 10^−5^
8	2.12 × 10^−4^	2.38 × 10^−4^	1.47 × 10^−4^	1.94 × 10^−4^	1.64 × 10^−4^	1.03 × 10^−5^	1.01 × 10^−5^
9	2.15 × 10^−4^	2.37 × 10^−4^	1.53 × 10^−4^	1.91 × 10^−4^	1.61 × 10^−4^	1.04 × 10^−5^	1.07 × 10^−5^
10	2.15 × 10^−4^	2.37 × 10^−4^	1.42 × 10^−4^	1.91 × 10^−4^	1.61 × 10^−4^	1.05 × 10^−5^	1.03 × 10^−5^
Average	2.09 × 10^−4^	2.38 × 10^−4^	1.47 × 10^−4^	1.89 × 10^−4^	1.67 × 10^−4^	1.04 × 10^−5^	1.03 × 10^−5^
Stand. deviation *σ*	4.50 × 10^−6^	1.50 × 10^−6^	4.41 × 10^−6^	6.74 × 10^−6^	4.10 × 10^−6^	1.00 × 10^−7^	3.00 × 10^−7^

**Table 10:** The modeling results for *L*_WARP_CALC_ and *L*_WEFT_CALC_ − RSKCu+Ni.

Meas.	*L_WARP_CALC_*	*L_WEFT_CALC_*
No.	(m)	(m)
1	2.00 × 10^−4^	2.13 × 10^−4^
2	2.08 × 10^−4^	2.13 × 10^−4^
3	2.08 × 10^−4^	2.13 × 10^−4^
4	2.07 × 10^−4^	2.18 × 10^−4^
5	2.07 × 10^−4^	2.23 × 10^−4^
6	2.09 × 10^−4^	2.24 × 10^−4^
7	2.11 × 10^−4^	2.26 × 10^−4^
8	2.13 × 10^−4^	2.24 × 10^−4^
9	2.16 × 10^−4^	2.21 × 10^−4^
10	2.16 × 10^−4^	2.18 × 10^−4^
Average	2.10 × 10^−4^	2.19 × 10^−4^
Stand. deviation *σ*	4.60 × 10^−6^	4.70×10^−6^

**Table 11:** The measurement results of the length of the warp *L_WARP_MEAS_* and the length of the line connecting the arcs in warp *a*-RSKCu+Ni.

Meas. No.	Measured Parts of the arc					*L*_warp_MEAS_	*a*
	(m)					(m)	(m)
1	4.56 × 10^−5^	4.06 × 10^−5^	3.78 × 10^−5^	4.13 × 10^−5^	4.81 × 10^−5^	2.13 × 10^−4^	2.13 × 10^−4^
2	4.47 × 10^−5^	3.99 × 10^−5^	3.72 × 10^−5^	4.29 × 10^−5^	4.90 × 10^−5^	2.14 × 10^−4^	2.13 × 10^−4^
3	4.39 × 10^−5^	4.12 × 10^−5^	3.76 × 10^−5^	4.10 × 10^−5^	5.00 × 10^−5^	2.14 × 10^−4^	2.14 × 10^−4^
4	4.49 × 10^−5^	4.15 × 10^−5^	3.73 × 10^−5^	4.11 × 10^−5^	4.87 × 10^−5^	2.14 × 10^−4^	2.13 × 10^−4^
5	4.60 × 10^−5^	4.04 × 10^−5^	3.80 × 10^−5^	4.14 × 10^−5^	4.86 × 10^−5^	2.14 × 10^−4^	2.13 × 10^−4^
Average	4.50 × 10^−5^	4.07 × 10^−5^	3.76 × 10^−5^	4.15 × 10^−5^	4.89 × 10^−5^	2.14 × 10^−4^	2.13 × 10^−4^
Stand. deviation *σ*	7.00 × 10^−7^	6.00 × 10^−7^	3.00 × 10^−7^	7.00 × 10^−7^	6.00 × 10^−7^	3.00 × 10^−7^	4.00 × 10^−7^

**Table 12:** The comparison of measurement and calculated results of the length of the warp-RSKCu+Ni.

Meas. No.	*L*_warp_MEAS_	*L*_warp_CALC_	*L*_warp_MEAS_/*L*_warp_CALC_
	(m)	(m)	(%)
1	2.13 × 10^−4^	2.00 × 10^−4^	106.50
2	2.14 × 10^−4^	2.08 × 10^−4^	102.88
3	2.14 × 10^−4^	2.08 × 10^−4^	102.88
4	2.14 × 10^−4^	2.07 × 10^−4^	103.38
5	2.14 × 10^−4^	2.07 × 10^−4^	103.38
Average	2.14 × 10^−4^	2.06 × 10^−4^	103.81
Stand. deviation *σ*	3.00 × 10^−7^	3.02 × 10^−6^	1.36

**Table 13:** The modeling results for RSKCu+Ni.

Parameter	Average	Standard Deviation
*L*_WARP_CALC_ (m)	2.10 × 10^−4^	4.58 × 10^−6^
*L*_WEFT_CALC_ (m)	2.39 × 10^−4^	1.50 × 10^−6^
*S*_WARP_ (m^2^)	6.82 × 10^−15^	1.80 × 10^−16^
*S*_WEFT_ (m^2^)	7.73 × 10^−15^	2.20 × 10^−16^
*S*_Inter_point_ (m^2^)	1.70 × 10^−16^	1.00 × 10^−17^
*S*_Effect_ (m^2^)	7.11 × 10^−15^	1.60 × 10^−16^
*S1*_WARP_Multi_ (m^2^)	4.88 × 10^−14^	1.07 × 10^−15^
*S2*_WEFT_Multi_ (m^2^)	4.30 × 10^−14^	2.60 × 10^−16^

**Table 14:** The measurement results of the resistance between two electrodes: Cu plate + (Cu plate, CerexCuIv4, RSKCu+Ni, or RSKCuIv4).

Distance between Electrodes (m)	Resistance (Ω)			
	Cu plate	RSKCuIv4	CerexCuIv4	RSKCu+Ni
1.00 × 10^−2^	6.5 × 10^−1^	6.9 × 10^−1^	6.4 × 10^−1^	6.3 × 10^−1^
2.00 × 10^−2^	1.2 × 10^−2^	1.2 × 10^−2^	1.2 × 10^−2^	1.1 × 10^−2^
3.00 × 10^−2^	1.7 × 10^−2^	1.6 × 10^−2^	1.6 × 10^−2^	1.5 × 10^−2^
4.00 × 10^−2^	2.0 × 10^−2^	1.9 × 10^−2^	1.9 × 10^−2^	1.8 × 10^−2^
5.00 × 10^−2^	2.2 × 10^−2^	2.1 × 10^−2^	2.1 × 10^−2^	2.1 × 10^−2^
10.00 × 10^−2^	3.1 × 10^−2^	3.1 × 10^−2^	3.2 × 10^−2^	3.0 × 10^−2^
			**Surface area (m^2^)**	
Average	3.06 × 10^−3^	3.18 × 10^−3^	3.22 × 10^−3^	3.28 × 10^−3^
Stand. deviation *σ*	0.00 × 10^0^	4.50 × 10^−5^	8.22 × 10^−5^	7.48 × 10^−5^

**Table 15:** The modeling results for surface area of samples.

Modeling	RSKCuIv4	CerexCuIv4	RSKCu+Ni
No.	(m^2^)	(m^2^)	(m^2^)
1	4.65 × 10^−3^	5.37 × 10^−3^	5.78 × 10^−3^
2	4.67 × 10^−3^	5.36 × 10^−3^	5.73 × 10^−3^
3	4.69 × 10^−3^	5.31 × 10^−3^	5.66 × 10^−3^
4	4.67 × 10^−3^	5.43 × 10^−3^	5.68 × 10^−3^
5	4.64 × 10^−3^	5.33 × 10^−3^	5.68 × 10^−3^
6	4.64 × 10^−3^	5.36 × 10^−3^	5.66 × 10^−3^
7	4.60 × 10^−3^	5.32 × 10^−3^	5.65 × 10^−3^
8	4.59 × 10^−3^	5.36 × 10^−3^	5.62 × 10^−3^
9	4.58 × 10^−3^	5.41 × 10^−3^	5.60 × 10^−3^
10	4.61 × 10^−3^	5.36 × 10^−3^	5.60 × 10^−3^
Average	4.63 × 10^−3^	5.36 × 10^−3^	5.67 × 10^−3^
Stand. deviation *σ*	3.62 × 10^−5^	3.54 × 10^−5^	5.23 × 10^−5^
